# Association of education level and depression with cognitive decline: findings from the examining cognitive health outcomes in heart failure study

**DOI:** 10.3389/fcvm.2025.1566400

**Published:** 2025-04-29

**Authors:** Marta Wleklik, Christopher S. Lee, Maria Jędrzejczyk, Heba Aldossary, Izabella Uchmanowicz

**Affiliations:** ^1^ Department of Nursing, Faculty of Nursing and Midwifery, Wroclaw Medical University, Wroclaw, Poland; ^2^ Boston College William F. Connell School of Nursing, Chestnut Hill, MA, United States; ^3^ Department of Nursing, Prince Sultan Military College of Health Sciences, Dhahran, Saudi Arabia; ^4^ Frances Payne Bolton School of Nursing at Case Western Reserve University, Cleveland, OH, United States; ^5^ Centre for Cardiovascular Health, Edinburgh Napier University, Sighthill Campus, Edinburgh, United Kingdom

**Keywords:** heart failure, cognitive impairment, depression, frailty syndrome, elderly

## Abstract

**Introduction:**

Cognitive decline in older adults with heart failure (HF) may be influenced by educational level and depressive symptoms. This study assesses the impact of these factors on cognitive function in this patient population to mitigate cognitive decline and improve overall health in this vulnerable population.

**Aim:**

To identify the predictors of cognitive impairment in older patients with heart failure using a longitudinal mixed-model analysis.

**Material and methods:**

A 250 HF patients aged 60 and older with an MMSE score ≥24 was evaluated. Cognitive function was assessed using the Mini-Mental State Examination (MMSE), mental health with the Hospital Anxiety and Depression Scale (HADS) and Patient Health Questionnaire-9 (PHQ-9), and nutritional status with the Mini Nutritional Assessment (MNA). Data were collected in three stages: baseline during hospitalization and at two subsequent hospital follow-ups. A linear mixed model analyzed the relationship between educational level, depressive symptoms, and MMSE scores, with a significance level set at *p* < 0.05.

**Results:**

The mean baseline MMSE score was 26.5 (SD = 2.1), suggesting good initial cognitive function among participants. Results from the linear mixed model indicated that each additional year of education correlated with a 0.161-point increase in MMSE scores (95%CI: 0.1–0.222, *p* < 0.001). Conversely, higher depressive symptoms were associated with poorer cognitive outcomes; specifically, each one-point increase in the HADS depression subscale corresponded to a 0.115-point decrease in MMSE scores (95%CI: −0.183 to −0.046, *p* = 0.001). Other factors, including age, sex, residence, and various comorbidities, did not show statistically significant associations with cognitive decline. At each stage of the study, approximately 8%, 11%, and 11% of patients, respectively, scored above the HADS cut-off for anxiety or depression, while an additional 13%, 12%, and 15% showed borderline scores. According to the PHQ-9, depressive symptoms of varying severity were present in 54% of patients at Stage I and II, and in 58% at Stage III.

**Conclusions:**

This study shows that greater educational background is associated with improved cognitive function, while higher levels of anxiety and depression are linked to cognitive decline in older adults with heart failure. These results highlight the importance of integrating mental health and education in interventions aimed at enhancing cognitive health in this population.

## Introduction

Heart failure (HF) is a common age-associated illness impacting around 64.3 million people worldwide (1). It signifies an advanced phase of several cardiovascular diseases, often associated with clinical decompensation that may require hospitalization but can be stabilized with appropriate treatment, allowing for patient discharge and follow-up (2). The incidence of heart failure is increasing, propelled by an aging global demographic, heightened population growth, and enhanced survival rates post-diagnosis. Consequently, in older patients, cardiovascular disease and frailty syndrome often co-occur, complicating clinical treatment and management (3).

The relationship between cardiovascular disease, cognitive impairment, and frailty is intricately complex and has substantial clinical implications. Frailty is a clinical illness marked by greater vulnerability and diminished functional reserves in response to an acute stressor. The most acknowledged definition is the Physical Frailty Phenotype, defined by Fried et al., which emphasizes five principal characteristics: unintentional weight loss, weakness, diminished endurance and energy, slowness, and reduced physical activity levels (4). Nonetheless, there is ongoing debate indicating that this definition may not adequately include other significant dimensions of frailty, especially cognitive function. The addition of cognitive elements has resulted in the emergence of the concept of cognitive frailty (5), a geriatric syndrome that markedly increases the risk of major adverse cardiovascular events, characterized by both the presence of physical frailty and cognitive impairment, excluding dementia (6).

Cognitive impairment may present in multiple cognitive domains, including memory, attention, and executive function, and it may or may not affect a patient's capacity to carry out daily tasks (7). It frequently acts as a precursor to dementia and exacerbates the vulnerability of persons already facing frailty. Studies demonstrate that cognitive impairment in frail individuals increases the predictive validity for negative health outcomes (8). Moreover, the occurrence of either geriatric syndrome heightens the probability of getting the other, as cerebral aging is a significant factor that might expedite physical frailty (8).

Although previous research has indicated a correlation between mental health, education, and cognitive function in older adults with HF, the longitudinal impact of these factors is still poorly understood (9–12). Therefore, this study aims to identify the predictors of cognitive impairment in older patients with heart failure using a longitudinal mixed-model analysis. By identifying these predictors and providing insights into the roles of education and mental health in protecting against cognitive impairment, the study seeks to inform interventions that will enhance cognitive outcomes in patients with HF.

## Material and methods

### Participants

The first stage of the study included 250 patients diagnosed with HF who had been hospitalized in a cardiology department due to a sudden deterioration of their condition. To be included in the study, participants had to be at least 60 years old, have been diagnosed with HF according to European Society of Cardiology (ESC) guidelines (13), have experienced heart failure for at least six months, be classified as New York Heart Association (NYHA) functional class II-IV, have been hospitalized for a HF episode and show good cognitive function as measured by the Mini-Mental State Examination (score of 24 or higher). Patients were excluded if they had NYHA class I, an MMSE score below 24 points, or a previously diagnosed and treated depressive disorder. Individuals with a history of anxiety disorders were also excluded. Patients without a formal psychiatric diagnosis but who scored above the clinical cut-off on the HADS or PHQ-9 were not excluded.

### Data collection

Patients were recruited from the Institute of Heart Diseases in University Hospital in Wroclaw, Poland, between September 2022 and July 2024. The study began with 250 participants. Data for Stage I were collected during hospitalization, following successful treatment of acute decompensated heart failure and after achieving clinical stability. Follow-up assessments were conducted approximately 6 months (Stage II; *n* = 162) and 12 months (Stage III; *n* = 124) after discharge during scheduled hospital visits. Participant attrition was due to death (*n* = 14) and loss to follow-up (*n* = 112). STROBE (Strengthening the Reporting of Observational Studies in Epidemiology) guidelines were followed.

### Research instruments

#### The mini nutritional assessment (MNA)

MNA was developed by a group of experts in the fields of nutrition, geriatrics, and medicine in 1994. The tool was created to assess the nutritional status of the elderly, enabling the identification of individuals at risk of malnutrition or who are malnourished, both in outpatient settings and in hospitals (14). It consists of two parts: a screening part and a detailed patient assessment. The screening part consists of 6 questions about weight changes, appetite, mobility, and overall health. The maximum score is 14 points. The results are interpreted as follows: 0–7 points indicates high risk of malnutrition; 8–11 points indicates possible risk of malnutrition; 12–14 points indicates no risk of malnutrition. For a more detailed assessment, the patient assessment part is used, which includes 12 specific questions regarding diet, skin condition, and medication use. The total score obtainable from both parts of the test is 30 points. The results are interpreted as follows: 24–30 points indicates good nutritional status; 17–23 points indicates risk of malnutrition; 0–16 points indicates malnutrition.

#### The mini-mental state examination (MMSE)

MMSE was developed by Folstein et al. in 1975 (15), is a commonly used tool to assess cognitive function across areas such as orientation, memory, attention, language, and executive skills. Scoring ranges up to 30 points, with scores below 24 suggesting potential cognitive impairment. The MMSE is valuable in both clinical and research settings for monitoring cognitive changes over time, especially in diagnosing dementia (16). A Polish version by Stanczak is widely used in Poland (17).

#### The hospital anxiety and depression scale (HADS)

HADS was developed in 1983 by Zigmond and Snaith to assess anxiety and depression levels in hospitalized patients (18). It includes 14 items, divided into two subscales: anxiety (HADS-A) and depression (HADS-D), with seven items each. Patients rate their symptoms over recent weeks on a 4-point scale (0–3), with higher scores indicating more severe symptoms. Each subscale has a total score ranging from 0 to 21, and a cut-off of 8/21 for both anxiety and depression was established by Bjelland et al (19). The study used the Polish adaptation of the HADS, validated by Mihalca and Pilecka (20).

#### The patient health questionnaire-9 (PHQ-9)

PHQ-9 is a self-report tool for measuring depression symptoms, developed by Kroenke and Spitzer. It is reliable and valid for assessing depression severity across various settings, including primary care (21). The questionnaire consists of nine core questions that evaluate the frequency of depressive symptoms over the past two weeks, such as depressed mood and sleep disturbances, plus an additional question about thoughts of death or self-harm. Each question is scored on a scale from 0 to 3, with higher scores indicating more severe symptoms. The study used the Polish adaptation of the PHQ-9, validated by Kokoszka et al (22). In this validation, a cut-off score of 12 points was determined to be optimal for screening depressive symptoms. This threshold is intended for screening purposes and does not equate to a clinical diagnosis, which must be confirmed by a qualified mental health professional.

### Statistical methods

The primary outcome variable, MMSE score, and several predictors were analyzed across multiple time points. Due to the repeated measures structure of the data, a linear mixed model was employed, with the patient identifier included as a random effect to account for intra-individual variability over time. This approach allowed the analysis of both fixed effects, such as education level and depressive symptoms (measured by HADS and PHQ-9), and random effects associated with individual patient differences. Model selection focused on the fixed effects of education (years) and HADS depression scores as predictors of cognitive performance, measured by changes in MMSE scores over time. The analysis specified a significance level of 0.05, interpreting *p*-values below this threshold as indicating statistically significant relationships. Calculations were conducted using R statistical software, version 4.4.1 (23).

## Results

Table 1 presents demographic and clinical data of patients collected across three stages of the study. The first stage includes data gathered during hospitalization following successful treatment of acute uncompensated heart failure, after achieving clinical stability. The second and third stages represent follow-up data collected during hospital controls. The table provides detailed information on patient characteristics such as age, education level, gender, marital status, place of residence, employment status, laboratory results, and comorbid conditions, as well as descriptive statistics from standardized questionnaires. This highlights variations in these parameters across the different stages of the study.

**Table 1:** Patient characteristics and data collection across three stages of the study.

Parameter		STAGE I (*N* = 250)	STAGE II (*N* = 162)	STAGE III (*N* = 124)
Age [years]	Mean (SD)	72.32 (6.73)	71.62 (6.42)	70.8 (6.2)
	Median (quartiles)	73 (67–76)	72 (67–75)	71 (66–75)
	Range	60–91	60–91	60–91
	*n*	250	162	124
Period of education [years]	Mean (SD)	12.26 (3.67)	12.45 (3.77)	12.64 (3.73)
	Median (quartiles)	11 (10–14)	12 (10–14)	12 (10–14)
	Range	7–27	7–27	7–27
	*N*	250	162	124
Sex	Woman	77 (30.80%)	49 (30.25%)	33 (26.61%)
	Man	173 (69.20%)	113 (69.75%)	91 (73.39%)
Marital status	Lonely	85 (34.00%)	55 (33.95%)	40 (32.26%)
	In a relationship	165 (66.00%)	107 (66.05%)	84 (67.74%)
Current place of residence	City	188 (75.20%)	126 (77.78%)	95 (76.61%)
	Village	62 (24.80%)	36 (22.22%)	29 (23.39%)
Professional status	Professionally active	31 (12.40%)	22 (13.58%)	18 (14.52%)
	Pensioner	219 (87.60%)	140 (86.42%)	106 (85.48%)
NT-proBNP [100 pg/ml]	Mean (SD)	38.82 (53.6)	38.28 (49.76)	34.64 (39.53)
	Median (quartiles)	16.54 (7.54–49.11)	21.78 (7.93–49.89)	18.88 (7.96–44.05)
	Range	0.95–389.22	1.26–389.22	1.26–182.85
	*n*	250	162	124
Previous heart attack	Mean (SD)	0.4 (0.49)	0.44 (0.5)	0.4 (0.49)
	Median (quartiles)	0 (0–1)	0 (0–1)	0 (0–1)
	Range	0–1	0–1	0–1
	*n*	250	162	124
Diabetes	Mean (SD)	0.55 (0.5)	0.57 (0.5)	0.55 (0.5)
	Median (quartiles)	1 (0–1)	1 (0–1)	1 (0–1)
	Range	0–1	0–1	0–1
	*n*	250	162	124
COPD/asthma	Mean (SD)	0.18 (0.39)	0.17 (0.37)	0.16 (0.37)
	Median (quartiles)	0 (0–0)	0 (0–0)	0 (0–0)
	Range	0–1	0–1	0–1
	*n*	249	161	124
Coronary artery disease	Mean (SD)	0.64 (0.48)	0.66 (0.48)	0.65 (0.48)
	Median (quartiles)	1 (0–1)	1 (0–1)	1 (0–1)
	Range	0–1	0–1	0–1
	*n*	250	162	124
Arterial hypertension	Mean (SD)	0.89 (0.32)	0.9 (0.3)	0.94 (0.23)
	Median (quartiles)	1 (1–1)	1 (1–1)	1 (1–1)
	Range	0–1	0–1	0–1
	*n*	250	162	124
Cardiovascular disease in the family	Mean (SD)	0.28 (0.45)	0.29 (0.46)	0.27 (0.44)
	Median (quartiles)	0 (0–1)	0 (0–1)	0 (0–1)
	Range	0–1	0–1	0–1
	*n*	250	162	124
Kidney disease	Mean (SD)	0.4 (0.49)	0.43 (0.5)	0.44 (0.5)
	Median (quartiles)	0 (0–1)	0 (0–1)	0 (0–1)
	Range	0–1	0–1	0–1
	*n*	250	162	124
Stroke/cerebrovascular disease	Mean (SD)	0.13 (0.34)	0.12 (0.33)	0.11 (0.32)
	Median (quartiles)	0 (0–0)	0 (0–0)	0 (0–0)
	Range	0–1	0–1	0–1
	*n*	250	162	124
Connective tissue diseases	Mean (SD)	0.22 (0.42)	0.23 (0.42)	0.25 (0.43)
	Median (quartiles)	0 (0–0)	0 (0–0)	0 (0–0.25)
	Range	0–1	0–1	0–1
	*n*	250	162	124
Cancers	Mean (SD)	0.17 (0.38)	0.14 (0.35)	0.15 (0.35)
	Median (quartiles)	0 (0–0)	0 (0–0)	0 (0–0)
	Range	0–1	0–1	0–1
	*n*	250	162	124
Peptic ulcer disease	Mean (SD)	0.12 (0.33)	0.12 (0.33)	0.13 (0.34)
	Median (quartiles)	0 (0–0)	0 (0–0)	0 (0–0)
	Range	0–1	0–1	0–1
	*n*	250	162	124
Liver diseases	Mean (SD)	0.1 (0.3)	0.09 (0.28)	0.1 (0.31)
	Median (quartiles)	0 (0–0)	0 (0–0)	0 (0–0)
	Range	0–1	0–1	0–1
	*n*	250	162	124
HF Group	HFrEF	105 (42.00%)	76 (46.91%)	58 (46.77%)
	HFmrEF	46 (18.40%)	31 (19.14%)	26 (20.97%)
	HFpEF	99 (39.60%)	55 (33.95%)	40 (32.26%)
NYHA Class	II	109 (43.60%)	69 (42.59%)	53 (42.74%)
	III	99 (39.60%)	66 (40.74%)	51 (41.13%)
	IV	42 (16.80%)	27 (16.67%)	20 (16.13%)
MNA [points]	Mean (SD)	24.19 (2.75)	24.69 (2.21)	24.78 (2.24)
	Median (quartiles)	24.5 (22.5–26)	25 (23–26.5)	25 (23–26.5)
	Range	13–32	16–28.5	19.5–29
	*n*	250	162	124
PHQ-9 [points]	Mean (SD)	5.9 (5.01)	5.94 (5.01)	6.44 (4.92)
	Median (quartiles)	5 (2–8)	5 (2–8)	6 (3–8,25)
	Range	0–27	0–23	0–23
	*n*	250	162	124
HADS: fear [points]	Mean (SD)	3.5 (3.41)	3.42 (3.68)	3.59 (3.43)
	Median (quartiles)	3 (1–5)	2 (0.25–5)	3 (1–6)
	Range	0–20	0–18	0–16
	*n*	250	162	124
HADS: depression [points]	Mean (SD)	2.76 (3.55)	3.25 (3.88)	3.88 (3.97)
	Median (quartiles)	1 (0–5)	2 (0–5)	3 (0–6)
	Range	0–16	0–20	0–17
	*n*	250	162	124
MMSE [points]	Mean (SD)	27.76 (1.85)	26.9 (1.74)	26.14 (1.67)
	Median (quartiles)	28 (26,25–29)	27 (26–28)	26 (25–28)
	Range	24–30	23–29	22–28
	*n*	250	162	124

SD, standard deviation; n, number of individuals; NT-proBNP, N-terminal pro B-type natriuretic peptide; HF- heart failure; HFrEF, heart failure with reduced ejection fraction; HFmrEF, heart failure with mildly reduced ejection fraction; HFpEF, heart failure with preserved ejection fraction; NYHA Class, New York Heart Association classification; MNA, mini nutritional assessment; PHQ-9, patient health questionnaire-9; HADS, Hospital Anxiety and Depression Scale; MMSE, Mini-Mental State Examination.

### Model

Table 2 presents the results of the multivariate linear mixed model, detailing the impact of education and depression on the MMSE scale scores. The data indicate that each additional year of education is associated with an average increase of 0.161 points on the MMSE scale. Conversely, each additional point on the HADS depression scale corresponds to an average decrease of 0.115 points on the MMSE scale.

**Table 2:** Results of the multivariate linear mixed model showing the effects of education and depression on MMSE scores.

Characteristic		Parameter	95%CI		p
Age [years]		−0.03	−0.064	0.004	0.087
Period of training [years]		0.161	0.1	0.222	<0.001*
Sex	Woman	ref.			
	Man	0.189	−0.34	0.719	0.482
Marital status	Lonely	ref.			
	In a relationship	0.334	−0.14	0.807	0.166
Current place of residence	City	ref.			
	Village	−0.192	−0.707	0.322	0.462
Professional status	Professionally active	ref.			
	Pensioner	0.026	−0.67	0.721	0.942
NT-proBNP [100 pg/ml]		0.001	−0.004	0.005	0.761
Previous heart attack		0.453	−0.062	0.968	0.084
Diabetes		0.147	−0.29	0.583	0.509
COPD/asthma		0.051	−0.507	0.609	0.857
Coronary artery disease		−0.001	−0.514	0.512	0.997
Arterial hypertension		0.229	−0.486	0.943	0.529
Cardiovascular disease in the family		−0.18	−0.666	0.307	0.467
Kidney disease		0.235	−0.216	0.686	0.305
Stroke/cerebrovascular disease		−0.209	−0.849	0.432	0.521
Connective tissue diseases		−0.095	−0.604	0.414	0.712
Cancers		0.139	−0.437	0.715	0.634
Peptic ulcer disease		−0.476	−1.135	0.183	0.156
Liver diseases		0.086	−0.65	0.822	0.817
HF Group	HFrEF	ref.			
	HFmrEF	0.298	−0.297	0.893	0.325
	HFpEF	−0.143	−0.676	0.39	0.596
NYHA Class	II	ref.			
	III	0.137	−0.27	0.544	0.509
	IV	−0.276	−0.787	0.236	0.29
MNA [pts]		−0.011	−0.079	0.056	0.744
PHQ-9 [pts]		0.015	-0.038	0.067	0.585
HADS: fear [pts]		0.023	-0.044	0.09	0.492
HADS: depression [pts]		−0.115	−0.183	−0.046	0.001*

NT-proBNP, N-terminal pro B-type natriuretic peptide; COPD, chronic obstructive pulmonary disease; HF- heart failure; HFrEF, heart failure with reduced ejection fraction; HFmrEF, heart failure with mildly reduced ejection fraction; HFpEF, heart failure with preserved ejection fraction; NYHA Class, New York Heart Association classification; MNA, mini nutritional assessment; PHQ-9, patient health questionnaire-9; HADS, Hospital Anxiety and Depression Scale; MMSE, Mini-Mental State Examination; 95%CI, 95% Confidence Interval; p, multivariate linear mixed model.

* statistically significant relationship (*p* < 0.05).

## Discussion

Cognitive impairment is common in patients with heart failure. Due to the complex mechanisms linking heart failure and cognitive disorders, factors characterizing this specific patient group are still being sought (24–27). The results of our study provide important information about the cognitive functioning of elderly patients with heart failure, as measured by the MMSE scale. Among the many sociodemographic and clinical factors assessed in this study, two had a significant impact on the MMSE score: the patients' education level and the severity of depressive symptoms, assessed using the HADS.

A lower level of education is identified as a risk factor for higher rates of mild cognitive impairment in patients with HF suggesting that education may play a key role in the severity of cognitive deficits observed in this patient population (24). In a study of outpatient HF patients aged ≥65 years, over 70% exhibited cognitive impairment, with significant differences depending on education level (28). In a cluster analysis of patients with chronic heart failure, about 34% were identified as having global cognitive deficits, and individuals with lower education were more frequently found in this group (29). In Cheng et al.'s study, 723 elderly individuals were included, and it was noted that cognitive impairment occurred in 54% of patients with less than 9 years of education, while it occurred in only 30% of those with 9 or more years of education (30). A multivariate logistic regression analysis showed that in patients with lower education levels, the odds ratio for cognitive impairment was significantly higher, at 2.5 (95% CI: 1.8–3.6), compared to individuals with higher education levels (30). In a more recent survey involving 605 patients with chronic stable heart failure across five European countries and the United States, the prevalence of cognitive impairment was found to be 67%. These studies suggest that the prevalence of mild cognitive impairment among heart failure patients is 1.5–2 times greater than that observed in the general population (31). These findings highlight the significant cognitive challenges faced by this patient population and underscore the need for further research to understand the underlying mechanisms and to develop targeted interventions.

Among the physiological and psychosocial factors linking education level and cognitive impairment, reduced cerebral blood flow caused by low cardiac output is cited as a significant mechanism contributing to cognitive deficits, particularly in individuals with lower education levels (24, 32). The severity of heart failure is also associated with cognitive decline. Patients in advanced NYHA classes exhibited greater cognitive deficits, including reduced memory and executive functions. This decline was more pronounced in individuals with lower education levels (24, 33). Additionally, elevated inflammatory markers are linked to decreased cognitive performance in HF patients (24, 32), as is the coexistence of multiple comorbidities, commonly observed in older individuals with heart failure. Multimorbidity may exacerbate the impact of low education on cognitive function, leading to poorer self-care and adherence to therapeutic recommendations, which are critical for managing both heart failure and cognitive health (34).

The results of our analysis indicate a positive effect of education on cognitive function. Specifically, each additional year of education increases the MMSE score by an average of 0.161 points. This finding aligns with existing literature, which has shown that a higher level of education may have a protective effect on cognitive function in older age. This effect can be explained by the concept of “cognitive reserve,” which refers to the brain's ability to compensate for neurodegenerative damage by more efficiently utilizing neural networks in individuals with higher education levels. Cognitive reserve may help mitigate the impact of brain aging and cardiovascular health on cognitive functions (33). Moreover, it suggests that individuals with higher education levels may better cope with brain pathology before clinical symptoms of cognitive decline appear (30, 35).

Research indicates that early-life education can significantly reduce the incidence of cognitive impairment and dementia. For example, individuals with higher education tend to show better cognitive performance and a slower rate of decline compared to those with lower education levels, even in the presence of brain pathology related to aging or cardiovascular issues (30, 36). Higher education may also be associated with a more intellectually active lifestyle, which could further delay cognitive decline in patients with heart failure. Late-life activities (mental, physical, and social) may mediate the relationship between early education and cognitive function. Engagement in these activities may further enhance cognitive reserve, providing additional protection and delaying cognitive decline in heart failure patients (36).

The results of this analysis also indicate that each additional point on the HADS depression scale lowers the MMSE score by an average of 0.115 points. This suggests that the severity of depressive symptoms is significantly associated with reduced cognitive function in patients with heart failure, a finding corroborated by literature. Cognitive impairment and depression commonly co-occur in HF patients. Studies show that up to 60% of HF patients experience depression, while depressive symptoms are linked to cognitive deficits in approximately 50% of cases (37). Notably, and alarmingly, in one study, nearly 48% of cognitive impairments and 52% of depressive symptoms were not recognized by clinicians (38). The interplay between depression and cognitive disorders in HF is significant and multifaceted. Depression is associated with physiological changes that can adversely impact cardiovascular outcomes, including increased sympathetic nervous system activity and elevated levels of inflammatory markers. These alterations can further complicate the clinical presentation of patients with HF (39–41). Moreover, depression may lead to decreased engagement in social and intellectual activities, which, in turn, exacerbates cognitive decline. Symptoms of depression can impair memory and executive functions, which are crucial for managing heart failure (37, 38). Additionally, the temporal trends in Table 1 support our findings. Over the study period, a gradual decline in MMSE scores and a concurrent increase in depressive symptoms (as measured by PHQ-9 and HADS-D) were noted. Although these changes were not subjected to additional statistical testing between time points, they may still reflect a clinically relevant pattern of cognitive and emotional deterioration post-discharge. This highlights the importance of long-term psychological and cognitive monitoring in this patient group, even after achieving clinical stability during hospitalization.

Cognitive impairment and depression are associated with increased morbidity and mortality in patients with heart failure (HF), underscoring the prognostic significance of cognitive function in the management of HF (37). Research has shown that a Montreal Cognitive Assessment (MoCA) score below 22 at discharge is linked to a sixfold increased risk of readmission due to HF and significantly predicts readmission for any cause (42). In a cohort study, it was found that the severity of depressive symptoms was a primary prognostic factor for health-related quality of life, overshadowing the impact of cognitive dysfunction (39).

While these results offer important insights, it is also important to consider the study's context. Although the statistical model adjusts for missing data, the natural reduction in participant numbers over time may have influenced the outcomes to a small extent. Those who remained in the study might have differed slightly in terms of health or engagement, which could have affected the observed relationships.

In summary, our study emphasizes the significance of education level and depressive symptoms as key factors influencing cognitive function in elderly patients with heart failure (HF). These findings suggest that both educational background and mental health should be considered in the context of the diagnosis and therapy of patients in this population. Further research should focus on a deeper understanding of the interactions between these variables and on effective interventions that could support cognitive function and mental health in older adults with heart failure.

### Clinical implications

The findings of this study have several clinical implications. First, they suggest that healthcare providers should integrate cognitive assessments into routine evaluations for older patients with heart failure, particularly focusing on education and mental health status. Early identification of cognitive impairment and depressive symptoms may facilitate timely interventions, such as cognitive training and mental health support, ultimately improving patient outcomes. Furthermore, educational programs tailored for both patients and caregivers could enhance understanding of the importance of cognitive health and strategies to mitigate decline. Additionally, mental health interventions, including counseling and pharmacological treatments, could play a vital role in supporting cognitive function among older adults with HF.

### Limitations of the study

While the results offer valuable insights, several limitations should be acknowledged. The sample size, although initially robust (*n* = 250), decreased across the stages of data collection, with only 124 participants completing the final follow-up. This attrition may introduce bias and limit the generalizability of the findings, as individuals who remained in the study could differ systematically from those who dropped out, particularly in terms of health status, psychological resilience, or access to social support. Moreover, due to the observational nature of the study, causal relationships between education, depression, and cognitive impairment cannot be definitively established. Future longitudinal research with larger, more diverse samples is needed to validate and expand upon these findings. Additionally, certain potentially influential variables such as medication adherence, physical activity, and the extent of social support were not assessed in this study. The absence of these measures may limit the explanatory scope of our findings, and future research should consider including these factors to better understand their role in cognitive outcomes among patients with heart failure.

## Conclusion

This study emphasizes the significant roles of education and mental health in cognitive outcomes among older adults with heart failure. The protective effect of educational attainment and the detrimental impact of depressive symptoms highlight the need for targeted interventions that address both cognitive and mental health aspects in this vulnerable population. By enhancing our understanding of these predictors, healthcare providers can better tailor interventions to improve cognitive health and overall quality of life for older adults living with heart failure. Further research is needed to elucidate the mechanisms underlying these associations and to explore additional factors that may contribute to cognitive impairment in this demographic.

## Data Availability

The raw data supporting the conclusions of this article will be made available by the authors, without undue reservation.
